# Erythromycin suppresses neutrophil extracellular traps in smoking-related chronic pulmonary inflammation

**DOI:** 10.1038/s41419-019-1909-2

**Published:** 2019-09-12

**Authors:** Hui Zhang, Shi-Lin Qiu, Qi-Ya Tang, Xiu Zhou, Jian-Quan Zhang, Zhi-Yi He, Jing Bai, Mei-Hua Li, Jing-Min Deng, Yi Liang, Xiao-Ning Zhong

**Affiliations:** Department of Respiratory and Critical Care Medicine, the First Affiliated Hospital of Guangxi Medical University, Nanning, China

**Keywords:** Inflammation, Respiratory tract diseases

## Abstract

Neutrophil extracellular traps (NETs) may play a critical role in smoking-related chronic airway inflammation. However, the mechanism by which NETs induced by cigarette smoke initiate the adaptive immunity in chronic obstructive pulmonary disease (COPD) is not fully understood. In this study, we explored the effects of NETs induced by cigarette smoke on the myeloid dendritic cells (mDCs) and Th1 and Th17 cells. Additionally, we observed the inhibitory effect of erythromycin on NETs induced by cigarette smoke. We found that elevated NET levels in the sputum of COPD patients were correlated with the circulating Th1 response, mDC activation and airflow limitation. NETs induced by cigarette smoke extract (CSE) could activate monocyte-derived mDCs and promote Th1 and Th17 differentiation in vitro. Erythromycin effectively inhibited NET formation induced by CSE. In vivo, erythromycin decreased NETs in the airway and ameliorated emphysema with Th1 and Th17 cell down-regulation and CD40^+^ and CD86^+^ mDCs suppression in mice chronically exposed to cigarette smoke. These findings provide direct evidence that NETs promote the differentiation of Th1 and Th17 and play a role in the adaptive immunity of smoking-related chronic lung inflammation. Erythromycin is a potential therapeutic strategy for NETs inhibition in COPD.

## Introduction

Chronic obstructive pulmonary disease (COPD) is a progressive disease associated with abnormal airway and alveolar inflammatory responses to cigarette smoke or other noxious particles and gases^1^. Owing to its high mortality and morbidity, COPD has become a serious global health issue^2^. Cigarette smoking is the major risk factor for COPD and directly promotes airway neutrophilic inflammation^3^. Interestingly, cigarette smoke extract (CSE) or nicotine can trigger neutrophil extracellular trap (NET) formation^4,5^. NETs were initially described as web-like DNA structures produced by activated neutrophils during the response to pathogens^6^. More recently, studies have indicated that NETs are involved in many non-infectious disorders and chronic inflammatory conditions, such as systemic lupus erythematosus^7^, autoimmune small-vessel vasculitis^8^, rheumatoid arthritis^9^, atherosclerosis^10^, and others^11–14^. Previous studies have revealed that excessive NETs in the sputum are associated with elevated pro-inflammatory cytokine levels and thus, might contribute to lung damage^15,16^. However, most studies have focused on the direct pathological effect of NETs^17,18^, and relatively little is known about the contribution of NETs to adaptive immunity.

The initiation and dysregulation of adaptive immunity are related, in part, to the enhancement and persistence of airway inflammation in COPD. We recently demonstrated that NETs released by activated polymorphonuclear neutrophils (PMNs) stimulated with CSE promote the activation of plasmacytoid dendritic cells (DCs) in mice and, subsequently initiate pathologic T cell responses^5^, suggesting that NETs serve as critical connectors between the innate and adaptive immunity and could serve as a potential target for COPD treatment. However, whether NETs induced by CSE could activate DCs in human remains to be elucidated.

Persistent neutrophilic infiltration in the airway is a typical feature of smoking-related COPD, which represents an obstacle for conventional therapeutic regimens. The anti-inflammatory and immuno-modulating effects of macrolides, such as erythromycin, have long been recognised. Emerging evidence has shown that long-term and low-dose erythromycin can inhibit airway neutrophilic inflammation and reduce acute exacerbations in patients with COPD^19^. Macrolides are now recommended by a series of clinical guidelines to prevent acute exacerbation of COPD^2,20^, but the mechanisms underlying their inhibitory effects on neutrophilic inflammation are still unclear. Several previous studies have reported that erythromycin could attenuate pulmonary inflammation, protect against the development of emphysema, and alter different types of pulmonary cells in murine models^21–24^. However, it is unclear whether erythromycin suppresses NET formation under stimulation with cigarette smoke and thus, modulates adaptive immunity.

Here, we sought to explore the effects of NETs induced by cigarette smoke on the differentiation of Th1 and Th17. We also observed the effect of erythromycin on NET formation during chronic inflammation induced by cigarette smoke exposure.

## Materials and Methods

Detailed methods are provided in the online supplement.

### Human blood and sputum specimen collection and preparation

Human blood and sputum samples were collected from 32 patients with COPD and 16 healthy controls. The diagnosis of COPD was based on forced expiratory volume in 1 s (FEV_1_) and forced vital capacity (FVC) detected by post-bronchodilator spirometry (FEV_1_ < 80% predicted and FEV_1_/FVC < 70%) established in the Global Initiative for Chronic Obstructive Lung Disease (GOLD)^24^. The grading of severity of airflow limitation in COPD was defined as: GOLD stage 1: FEV_1 _≥ 80% predicted; GOLD stage 2: 50% ≤ FEV_1 _< 80% predicted; GOLD stage 3: 30% ≤ FEV_1 _< 50% predicted; GOLD stage 4: FEV_1 _< 30% predicted^24^.

Peripheral blood mononuclear cells (PBMCs) were separated by Lymphoprep (Stemcell Technologies, Canada) centrifugation. Th1 (CD4^+^ IFN-γ^+^ T) and Th17 (CD4^+^ IL-17^+^ T) cells as well as CD40^+^ and CD86^+^ myeloid dendritic cells (mDCs) in PBMCs were analysed by flow cytometry. In some experiments, PBMCs of healthy donors were used to generate monocyte-derived mDCs or to isolate naïve CD4^+^ T lymphocytes. PMNs in the blood were isolated as described previously^25^. Sputum induction was performed as previously described^26^. Cell-free extracellular DNA in sputum was detected by using the Quant-iT PicoGreen dsDNA Assay Kit (Invitrogen, Carlsbad, CA, USA) following the manufacturer’s instructions.

All the subjects provided informed consents, and the Ethics Committee of the First Affiliated Hospital of Guangxi Medical University granted ethical approval.

### Animals

Thirty male C57BL/6J mice (8 weeks old) were randomly divided into three groups: Air group, cigarette smoke (CS) group, and erythromycin (EM) group (*n* = 10/group). The CS group and EM group were exposed to cigarette smoke for 24 weeks as previously described^27^. The Air group was exposed to room air for 24 weeks. In the EM group, erythromycin (Sigma-Aldrich, St. Louis, MO, USA) was orally administered at 100 mg/kg/d from the 12th week of cigarette exposure. In the CS and Air group, an identical volume of vehicle was administered orally. At the end of the 24th week, mice were anaesthetised and sacrificed.

PMNs were isolated using peripheral blood neutrophil separation medium for mouse (TBD, Tianjin, China). Bronchoalveolar lavage fluid (BALF) was collected. Extracellular DNA in BALF was detected by PicoGreen. The total cell count in the BALF was determined in a Bürcker chamber, and the neutrophil cell count was obtained after Wright’s Giemsa staining. The severity of emphysema was assessed by the mean linear intercept (MLI). Single-cell suspensions from the lungs were prepared for flow cytometry as described previously^28^. All mice were purchased from the Guangxi Medical University Laboratory of Animal Centre and all animal experiments in this study were approved by the Laboratory Animal Ethics Committee of Guangxi Medical University (Nanning, China).

### Immunofluorescence of NETs

NETs were stained for DNA with propidium iodide (PI; Sigma-Aldrich) or 4′,6-diamidino-2-phenylindole dihydrochloride (DAPI; Sigma-Aldrich). In some experiment, neutrophil elastase (NE), myeloperoxidase (MPO), and citrullinated histone H3 (CITH3) on NETs were observed by immunofluorescence staining.

### CSE-induced NETs assay

CSE was prepared as described previously^29^. Freshly isolated PMNs from the blood of COPD patients or mice with emphysema were seeded in 24-well culture plates (1.0 × 10^6^ cells) with serum-free RPMI 1640. PMNs were stimulated with 0.3% CSE for 4 h, with or without pre-treatment with erythromycin (10 μg/mL for human samples; 2 μg/mL for mice) for 30 min. Phorbol-12-myristate-13-acetate (PMA, 100 nmol/L = 61.68 μg/mL; Sigma-Aldrich) and diphenyl iodine (DPI; Sigma-Aldrich) were used in some of the experiments as positive controls for stimulating and inhibiting NETs, respectively. Cell-free extracellular DNA in supernatants was detected using PicoGreen.

For NE and MPO concentration assay, PMNs (2.0 × 10^6^ cells) were seeded in 24-well culture plates and stimulated with 0.3% CSE for 4 h with or without pre-treatment of erythromycin for 30 min. NET-associated NE and MPO were detected using the NETosis Assay kit (Cayman Chemical, Ann Arbor, MI, USA) and MPO ELISA kit (Cusabio, Wuhan, China), respectively.

For the intracellular reactive oxygen species (ROS) production assay, PMNs (2.0 × 10^6^ cells) of patients with COPD were seeded in 24-well culture plates and stimulated with 0.3% CSE for 1 h in a CO_2_ incubator at 37 °C with or without pre-treatment of erythromycin or DPI for 30 min. After stimulation, PMNs were harvested and further incubated with 1 μmol/L ROS probe 2′,7′-dichlorofluorescein diacetate (DCFH-DA; Sigma-Aldrich) at 37 °C for 30 min. The DCFH-DA stained PMNs were detected by flow cytometry immediately.

### Preparation of monocyte-derived mDCs

Monocytes isolated by adherence from PBMCs of healthy donors were cultured with RPMI 1640 containing 10% FBS, 1000 IU/mL granulocyte-macrophage colony stimulating factor (GM-CSF; Peprotech, Rocky Hill, USA) and 500 IU/mL IL-4 (Peprotech) for 6 days as previously described^30^. At day 6, immature mDCs were harvested and isolated by positive selection (CD209 (DC-SIGN) MicroBead Kit, human, Miltenyi Biotec, Aubum, CA).

### Preparation of CSE-induced NETs and stimulation of mDCs

PMNs from patients with COPD were stimulated with 0.3% CSE for 4 h. CSE-induced NETs were collected as previously described^5^. To determine the effect of CSE-induced NETs on mDCs maturation, the purified mDCs were plated in 6-well plates at a density of 1.0 × 10^6^ cells/mL in the presence or absence of CSE-induced NETs (20 ng/mL) for 15 h. Subsequently, CD11c, CD40, CD86, and HLA-DR on the surface of mDCs were analysed by flow cytometry. The supernatants of mDCs were collected and detected IL-1β, IL-12, and TNF-α by ELISA.

In some experiments, mDCs were generated for assessing the capacity to promote the differentiation of naïve CD4^+^ T lymphocytes. The purified immature mDCs of day 6 were primed with IFN-γ (10 ng/mL; Peprotech) until day 7. Then the primed mDCs were stimulated with or without NETs (20 ng/mL) for 24 h. CD4^+^ naïve T cells isolated by negative selection (Naive CD4^+^ T Cell Isolation Kit II human, Miltenyi) from PBMCs isolated from blood of healthy donors were cultured with mDCs (pre-treated with or without NETs) at a DC/T cell ratio of 1:5 for 4 days in 24-well plates. Cells were stimulated with 50 ng/mL PMA and 1 µg/mL ionomycin in the presence of GolgiStop (BD Pharmingen, San Diego, CA, USA) for 5 h. Cells were then collected and stained for CD4, IFN-γ and IL-17A which were detected by flow cytometry.

### Flow cytometry

The detection of surface molecule, intracellular cytokine, and intracellular ROS were performed by flow cytometry. Detailed methods are provided in the online supplementary file.

### ELISA

The IL-1β, IL-12, and TNF-α concentration in supernatants of mDCs and the soluble NE, MPO, and CITH3 levels in BALF of mice were measured by ELISA (IL-1β, IL-12, TNF-α, and MPO: Cusabio; NE: USCN Life Sciences, Wuhan, China; CITH3: Cayman Chemical) following the manufacturer’s instructions.

### Statistical analysis

Results were expressed as medians. Comparisons between two groups were evaluated using Mann–Whitney test. Comparisons between three or more groups were evaluated using Kruskal–Wallis one-way ANOVA on ranks. The correlation analyses were performed using Spearman’s rank correlation coefficient. Analyses were implemented in SPSS version 17.0 and *P* *<* 0.05 was considered significant.

## Results

### Characteristics of participants

The characteristics of the 32 patients with COPD and the 16 healthy controls are shown in Table 1.

**Table 1:** Characteristics of patients and healthy controls

Characteristics	Healthy controls (*n* = 16)	COPD (*n* = 32)
Age (year)	64.5 ± 9.0	68.5 ± 9.71
Male/Female	14/2	31/1
Current Smokers	0	31
Smoking history (pack years)	0	31.8 ± 11.8
BMI (kg/m^2^)	22.8 ± 2.4	22.5 ± 3.7
FEV_1_/FVC (%)	82.7 ± 7.5	53.7 ± 13.6*^,^**
FEV_1_ (%pred)	88.9 ± 7.4	44.4 ± 19.2*^,^**
GOLD Stage 1	N/A	3
GOLD Stage 2	N/A	9
GOLD Stage 3	N/A	10
GOLD Stage 4	N/A	10
Medications		
ICS	N/A	32
LABA	N/A	32
LAMA	N/A	9
Theophyline	N/A	10
Systemic steroids	N/A	0
Antimicrobials	N/A	0
Sputum cell counts		
Total cells (10^6^/ml)	0.67 (0.36–0.91)	2.28 (0.96–3.56)*^,^**
Neutrophils (10^6^/ml)	0.15 (0.07–0.25)	0.98 (0.38–1.52)*^,^**
Macrophages (10^6^/ml)	0.45 (0.27–0.72)	1.13 (0.50–1.92)*^,^**
Lymphocytes (10^6^/ml)	0.04 (0.01–0.06)	0.24 (0.08–0.44)*^,^**

Data are expressed as means ± SD or medians (range). Statistical analysis was performed with Kruskal–Wallis one-way ANOVA on ranks *p *<* *0.05* when compared with non-smokers. **p *<* *0.05* when compared with healthy smokers

*M* male, *F* female, *BMI* body mass index, *FEV*_*1*_ forced expiratory volume in 1 s, *FVC* forced vital capacity, *FEV*_*1*_*(% pred)* the ratio of actual FEV_1_ to predicted FEV_1_, *GOLD* Global Initiative for Chronic Obstructive Lung Disease, *LCS* inhaled corticosteroids, *LABA* long-acting β-agonist, *LAMA* long-acting muscarinic agonist, *N/A* not applicable

### NETs are abundantly produced in the sputum of patients with COPD

**Fig. 1:**
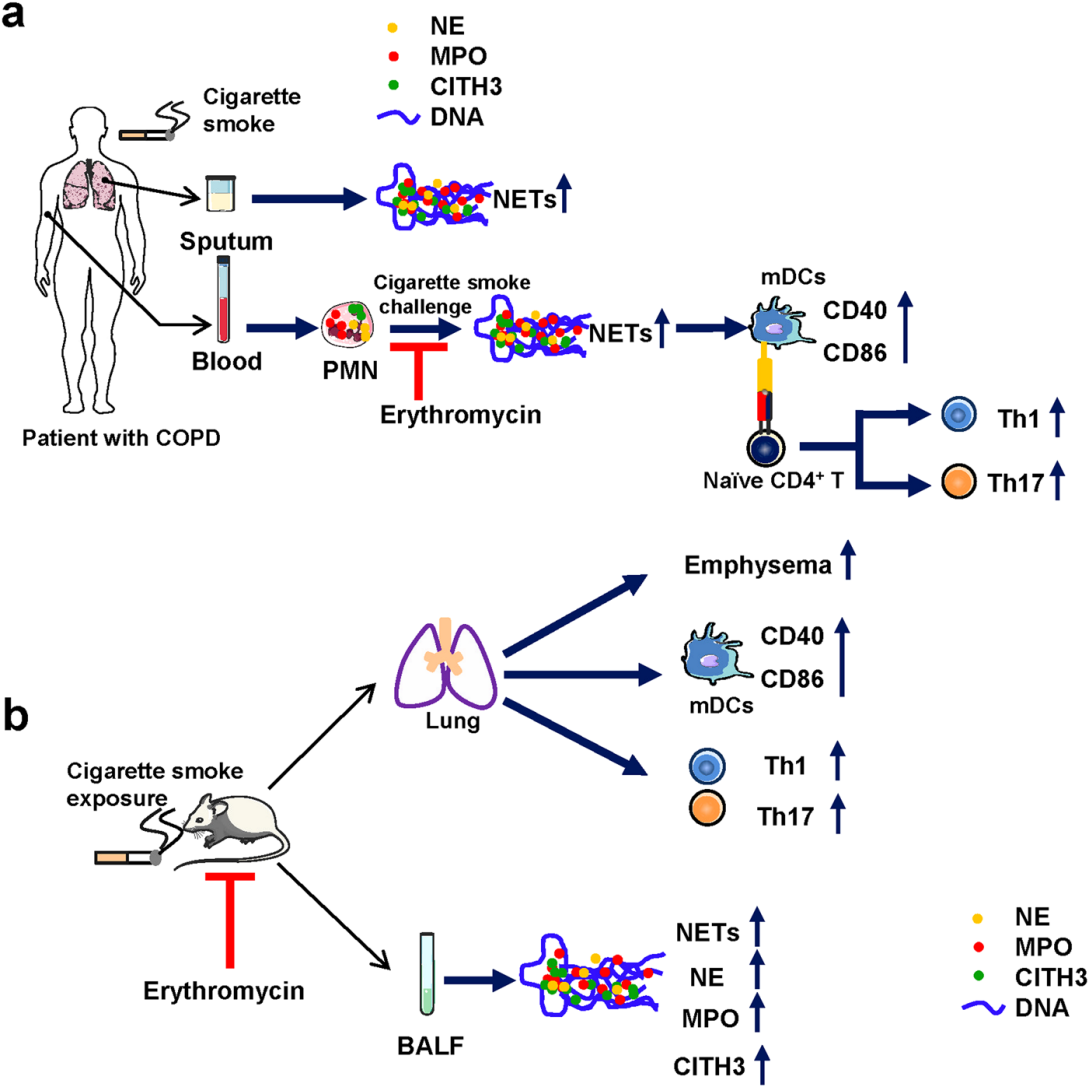
The flow diagram of experimental design. **a** The flow diagram of experimental design of human part which is to demonstrate the increased NETs, the activation of mDCs, and the enhanced Th1/Th17 cell responses in COPD patients; NETs promote mDCs activation and Th1/Th17 differentiation; Erythromycin inhibits NETs in human *vitro*. PMN polymorphonuclear neutrophil, NETs neutrophil extracellular traps, NE neutrophil elastase, MPO myeloperoxidase, CITH3 citrullinated histone H3, mDCs myeloid dendritic cells, Th1 Helper T lymphocytes 1, Th17 Helper T lymphocytes 17. **b** The flow diagram of experimental design of animal part which is to demonstrate that erythromycin inhibits NETs and attenuates the activation of mDCs and Th1/Th17 cell responses in a murine model of emphysema in vivo. BALF bronchoalveolar lavage fluid, CITH3, citrullinated histone H3

A flow diagram of the experimental design of this study is shown in Fig. 1.

**Fig. 2:**
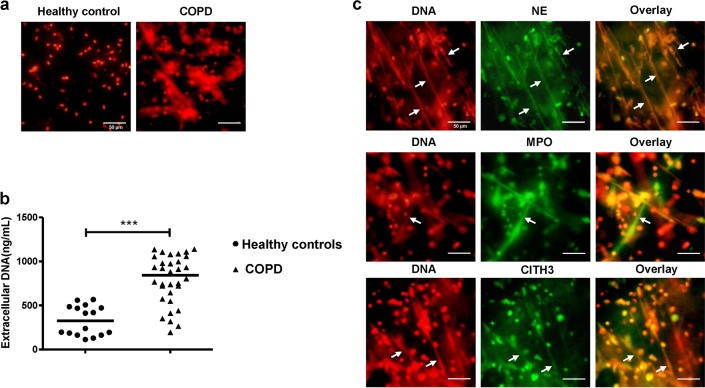
Excessive NETs in sputum of COPD. **a** The immunofluorescence of spontaneous NETs in sputum of COPD and healthy controls. The diluted sputum was seeded on poly-d-lysine-coated coverslips in 24-well round-bottom culture plates and allowed to settle for 1 h without stimulation. Cells on coverslips were fixed with 4% paraformaldehyde and permeabilised with 0.5% Triton X-100. To visualise the backbone of NETs, DNA was stained with propidium iodide (PI) and examined by fluorescence microscopy. Original magnification, ×400. Scale bars = 50 µm. **b** Concentrations of extracellular DNA in sputum of healthy controls (*n* = 16) and COPD (*n* = 32) were detected by PicoGreen fluorescence quantitative assay. Data are expressed as medians. The comparisons were determined by Mann–Whitney test. ***P *<* 0.001. **c** The diluted sputum of patient with COPD was seeded on poly-d-lysine-coated coverslips in 24-well round-bottom culture plates and allowed to settle for 1 h without stimulation. Cells on coverslips were fixed with 4% paraformaldehyde and permeabilised with 0.5% Triton X-100. NETs in sputum of COPD were stained with NE, MPO, or CITH3 (green) and extracellular DNA was stained with propidium iodide (PI, red) (NETs: white arrows). Original magnification, ×400. Scale bars = 50 µm

We first observed NETs in the sputum of healthy controls and patients with COPD. Under the fluorescence microscopy, the patients with COPD presented a considerable amount of spontaneous NETs in sputum (Fig. 2a). As determined by PicoGreen, the extracellular DNA concentration in the sputum was markedly higher in individuals with COPD than in healthy controls (*P* *<* 0.001; Fig. 2b). In addition, we observed that NE, MPO, and CITH3 were embedded in the network structure of extracellular DNA in the sputum of COPD patients (Fig. 2c).

### PMNs isolated from COPD patients are more sensitive to CSE

**Fig. 3:**
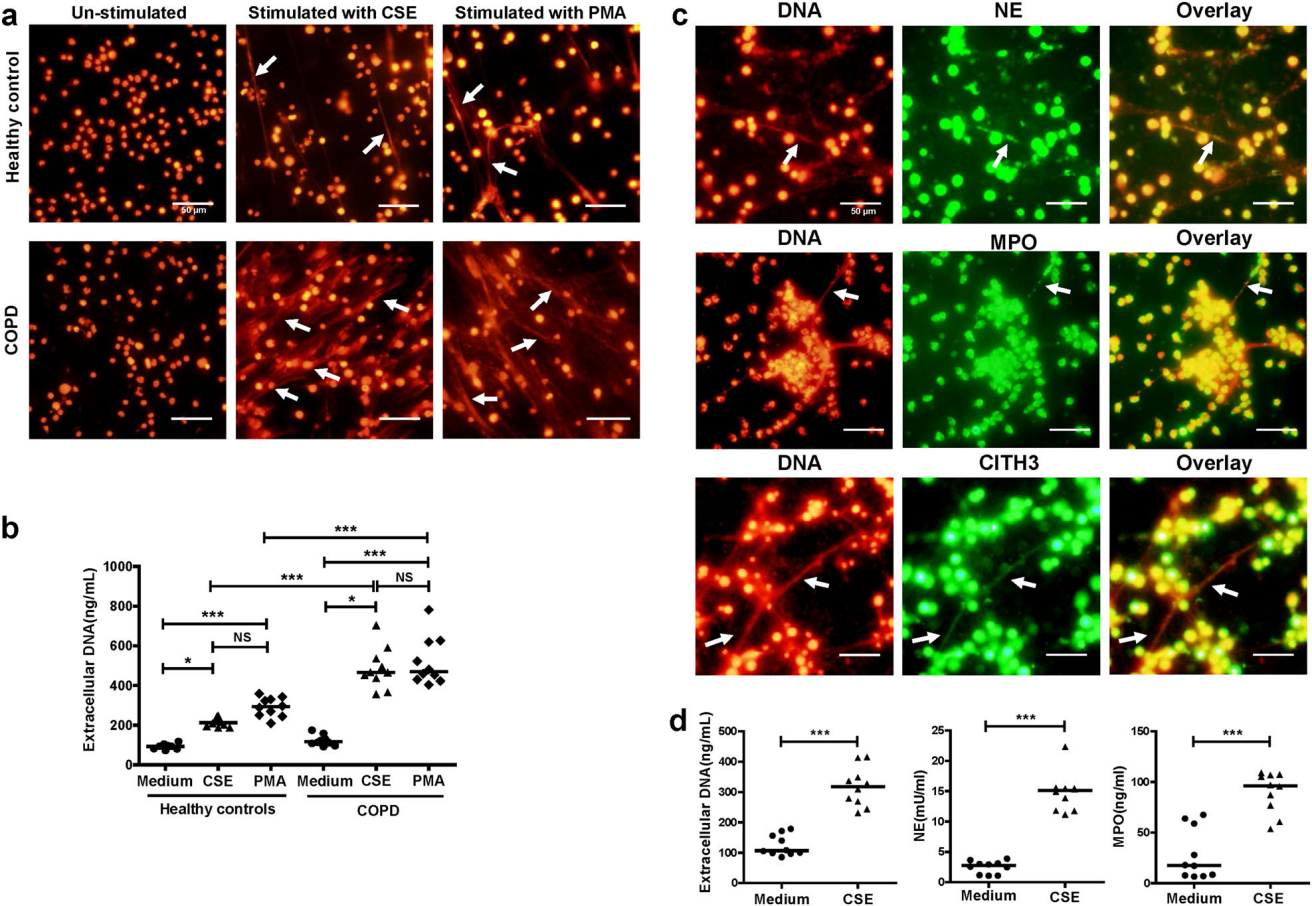
CSE induces more NETs in PMNs of COPD. **a** PMNs of healthy controls and COPD patients were stimulated with 0.3% cigarette smoke extract (CSE) for 4 h at 37 °C. Then neutrophils were fixed with 4% paraformaldehyde. The extracellular DNA was stained with PI (NETs: white arrows). Original magnification, ×400. Scale bars = 50 µm. **b** Concentrations of extracellular DNA in supernatants of CSE-induced PMNs and PMNs without stimulation (Medium) were investigated by PicoGreen fluorescence quantitative assay. Data are expressed as medians (*n* = 10). The comparisons were determined by Kruskal–Wallis one-way ANOVA on ranks. *P *<* 0.05, ***P *<* 0.001; NS not significant. **c** PMNs of patients with COPD were stimulated with 0.3% CSE for 4 h at 37 °C, and were fixed with 4% paraformaldehyde. Then components of NETs were stained with NE, MPO or CITH3 (green), and extracellular DNA stained with PI (red) (NETs: white arrows). Original magnification, ×400. Scale bars = 50 µm. **d** PMNs of patients with COPD were stimulated with 0.3% CSE for 4 h at 37 °C. Concentrations of extracellular DNA in supernatants of PMNs stimulated by CSE and PMNs without stimulation (Medium) were detected by PicoGreen fluorescence quantitative assay. Quantification of NET-associated NE and MPO were detected using the NETosis Assay kit and MPO ELISA kit. Data are expressed as medians (*n* = 10). The comparisons were determined by Mann–Whitney test. ***P < 0.001

We previously observed that PMNs of mice with emphysema seem to be highly sensitive to NET production^16^. To investigate whether PMNs of COPD were more susceptible to NET formation than those of healthy controls, we used 0.3% CSE to trigger NET formation in the blood PMNs of healthy controls and COPD patients and observed NET formation (Fig. 3a). As determined by PicoGreen, the PMNs from both COPD patients and healthy controls produced more extracellular DNA under the stimulation of CSE than their un-stimulated controls (Both *P* *<* 0.05; Fig. 3b). Notably, The PMNs from COPD patients produced more extracellular DNA than those from healthy controls under the stimulation of CSE (*P* *<* 0.001; Fig. 3b).

We observed NE, MPO, and CITH3 in the network structure of CSE-induced NETs in the blood of COPD patients by immunofluorescence (Fig. 3c). We also quantitatively analysed the extracellular DNA, and NET-associated NE and MPO released by CSE-stimulated PMNs from COPD patients. The levels of extracellular DNA, as well as NET-associated NE and MPO were significantly increased by CSE stimulation (All *P* *<* 0.001; Fig. 3d).

### Correlations between NETs in sputum, Th1/Th17 responses, DCs activation, and the severity of COPD

**Fig. 4:**
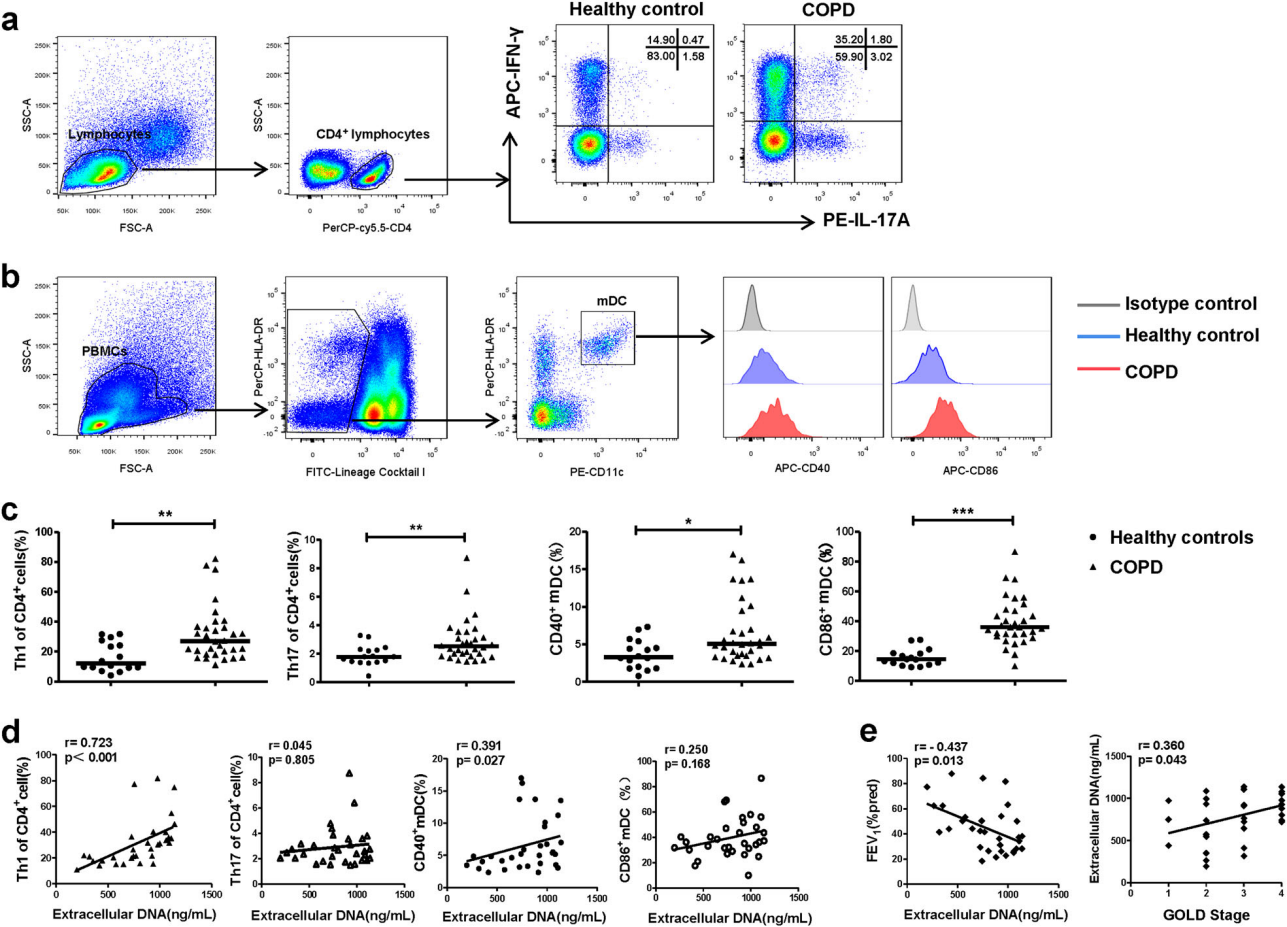
Correlations between extracellular DNA in the sputum, and Th1/Th17 responses, and the activation of DCs in COPD. **a** Peripheral bloods of mononuclear cells (PBMCs) of 16 healthy controls and 32 COPD patients were isolated by Lymphoprep centrifugation and detected by flow cytometry. Lymphocytes of PBMCs were identified based on forward-scattered light (FSC) and side-scattered light (SSC). Th1 cells were identified by CD4^+^IFN-γ^+^ cells and Th17 cells were identified by CD4^+^IL-17^+^ cells. Representative scatter plots of Th1 cells and Th17 cells of healthy controls and patients with COPD were shown. **b** PBMCs were identified based on FSC and SSC. The mDCs were identified by CD11c^+^HLA-DR^high^ cells in FITC-Lin^dim/negative^ cells. Representative histograms of CD40 and CD86 of mDCs in healthy controls and COPD were shown. **c** The proportions of Th1 and Th17 cells in CD4^+^ cells and the proportions of CD40^+^ and CD86^+^ mDCs in PBMCs of 16 healthy controls and 32 patients with COPD. Data are expressed as medians. The comparisons were determined by Mann–Whitney test. *P *<* 0.05, **P *<* 0.01, ***P *<* 0.001. **d** The correlations between extracellular DNA in sputum of COPD patients and circulating Th1 and Th17 cells and CD40 and CD86 on mDCs in PBMCs were determined by Spearman’s rank correlation coefficient. **e** The correlations between extracellular DNA in sputum of COPD patients and the ratio of actual FEV_1_ to predicted FEV_1_ (FEV_1_% pred), and GOLD stage were determined by Spearman’ s rank correlation coefficient

We observed Th1 and Th17 cells as well as the costimulatory molecules CD40 and CD86 on mDCs in PBMCs by flow cytometry (Fig. 4a, b). Th1 and Th17 cells were more prominent in COPD patients than in healthy controls (both *P* *<* 0.01; Fig. 4c). Moreover, circulating mDCs in patients showed a more activated phenotype with elevated expression of CD40 and CD86 (*P* *<* 0.05 and *P* *<* 0.001, respectively; Fig. 4c).

Next, we assessed the correlations between NETs in the sputum, and circulating Th1 and Th17 responses, and the activation of mDCs in COPD patients. Th1 cells and CD40 on mDCs were positively correlated with extracellular DNA in the sputum (*r* = 0.723, *P* *<* 0.001; *r* = 0.391, *P* = 0.027, respectively; Fig. 4d). However, there was no correlation between the frequencies of circulating Th17 cells or CD86 and the levels of extracellular DNA in sputum (*r* = 0.045, *P* = 0.805; *r* = 0.250, *P* = 0.168, respectively; Fig. 4d).

Moreover, we found that extracellular DNA in the sputum was negatively correlated with percent predicted FEV_1_ (*r* = −0.437, *P* *=* 0.013), and positively correlated with GOLD stage (*r* = 0.360, *P* *=* 0.043; Fig. 4e). However, extracellular DNA in sputum did not correlate with age, smoking pack years or body mass index in patients with COPD (Table 2).

**Table 2:** Correlations of NETs with clinical indicators in patients with COPD

Variable	*R* value	*P* value
Age (year)	0.0244	0.8945
Smoking history (pack years)	0.1303	0.4770
BMI (kg/m^2^)	−0.0500	0.7854
FEV_1_/FVC (%)	−0.4018	0.0227
FEV_1_ (%pred)	−0.4366	0.0125
GOLD Stage	0.3601	0.0429

Statistical analysis was performed with Spearman’s rank correlation coefficient. BMI, body mass index; FEV_1_, forced expiratory volume in 1 s

*FVC* forced vital capacity, *FEV*_*1*_*(% pred)* the ratio of actual FEV_1_ to predicted FEV_1_, *GOLD* Global Initiative for Chronic Obstructive Lung Disease

### NETs induced by CSE promote the activation of mDCs and the generation of Th1 and Th17 cells in vitro

**Fig. 5:**
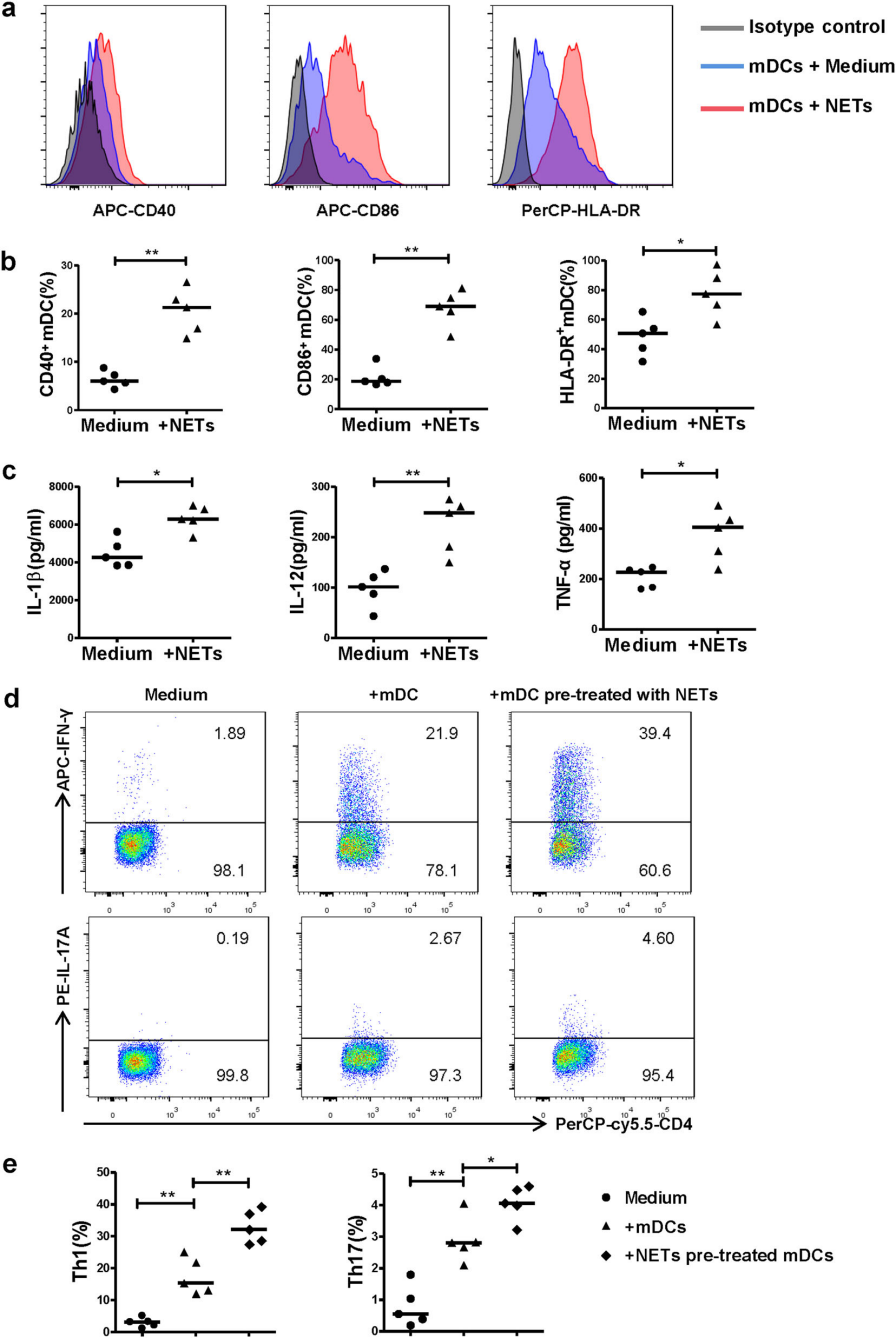
CSE-induced NETs promote the activation of mDCs and the generation of Th1 and Th17 cells. **a** PMNs isolated from patients of COPD were stimulated with 0.3% CSE for 4 h. CSE-induced NETs were harvested by AluI. The purified day 6 monocyte-derived mDCs of healthy donors were stimulated with CSE-induced NETs for 15 h and detected by flow cytometry. Representative histograms of CD40, CD86, and HLA-DR gated from mDCs were shown. **b** The CD40, CD86 and HLA-DR expression on mDCs with or without CSE-induced NETs stimulation were detected by flow cytometry. Data are expressed as medians (*n* = 5). The comparisons were determined by Mann–Whitney test. *P *<* 0.05, **P *<* 0.01. **c** The concentrations of IL-1β, IL-12 and TNF-α in supernatants of mDCs with or without CSE-induced NETs stimulation were detected by ELISA. Data are expressed as medians (*n* = 5). The comparisons were determined by Mann–Whitney test. *P *<* 0.05. **d** The purified immature mDCs of day 6 were primed with IFN-γ (10 ng/mL) for 24 h, and stimulated with CSE-induced NETs (20 ng/mL) for another 24 h. CD4^+^ naïve T cells isolated by negative selection from PBMCs of healthy donors were cultured with mDCs (stimulated with or without NETs) at a DC/T cell ratio of 1:5 for 4 days. Th1 and Th17 in the co-culture condition were detected by flow cytometry. Representative scatter plots of Th1 cells and Th17 cells in the co-culture condition were shown. **e** The propotions of Th1 cells and Th17 cells in the co-culture condition were detected by flow cytometry. Data are expressed as medians (*n* = 5). The comparisons were determined by Mann–Whitney test. *P *<* 0.05, **P *<* 0.01

To test if NETs induced by CSE could directly contribute to the activation of mDCs and promote the generation of Th1 and Th17, we isolated and used CSE-induced NETs to simulate mDCs, and then co-cultured CD4^+^ naïve T cells with mDCs pre-treated with or without NETs. As expected, mDCs stimulated with CSE-induced NETs for 15 h expressed higher levels of CD40, CD86 and HLA-DR than un-stimulated mDCs (*P* *<* 0.01, *P* *<* 0.01 and *P* *<* 0.05, respectively; Fig. 5a, b). Meanwhile, the levels of IL-1β, IL-12, and TNF-α were higher in the supernatant of mDCs stimulated with CSE-induced NETs than in those without NETs stimulation (*P* *<* 0.05, *P* *<* 0.01 and *P* *<* 0.05, respectively; Fig. 5c).

Regarding antigen-specific T-cell response, CD4^+^ naïve T cells cultured with mDCs pre-treated with NETs generated more Th1 and Th17 cells than those cultured with un-stimulated mDCs (*P* *<* 0.01 and *P* *<* 0.05, respectively; Fig. 5d, e).

### Erythromycin effectively inhibits NET formation and ROS production induced by CSE in vitro

To evaluate the effects of erythromycin on NET formation, we pre-treated human and mice neutrophils with erythromycin before stimulating NET formation with CSE in vitro.

**Fig. 6:**
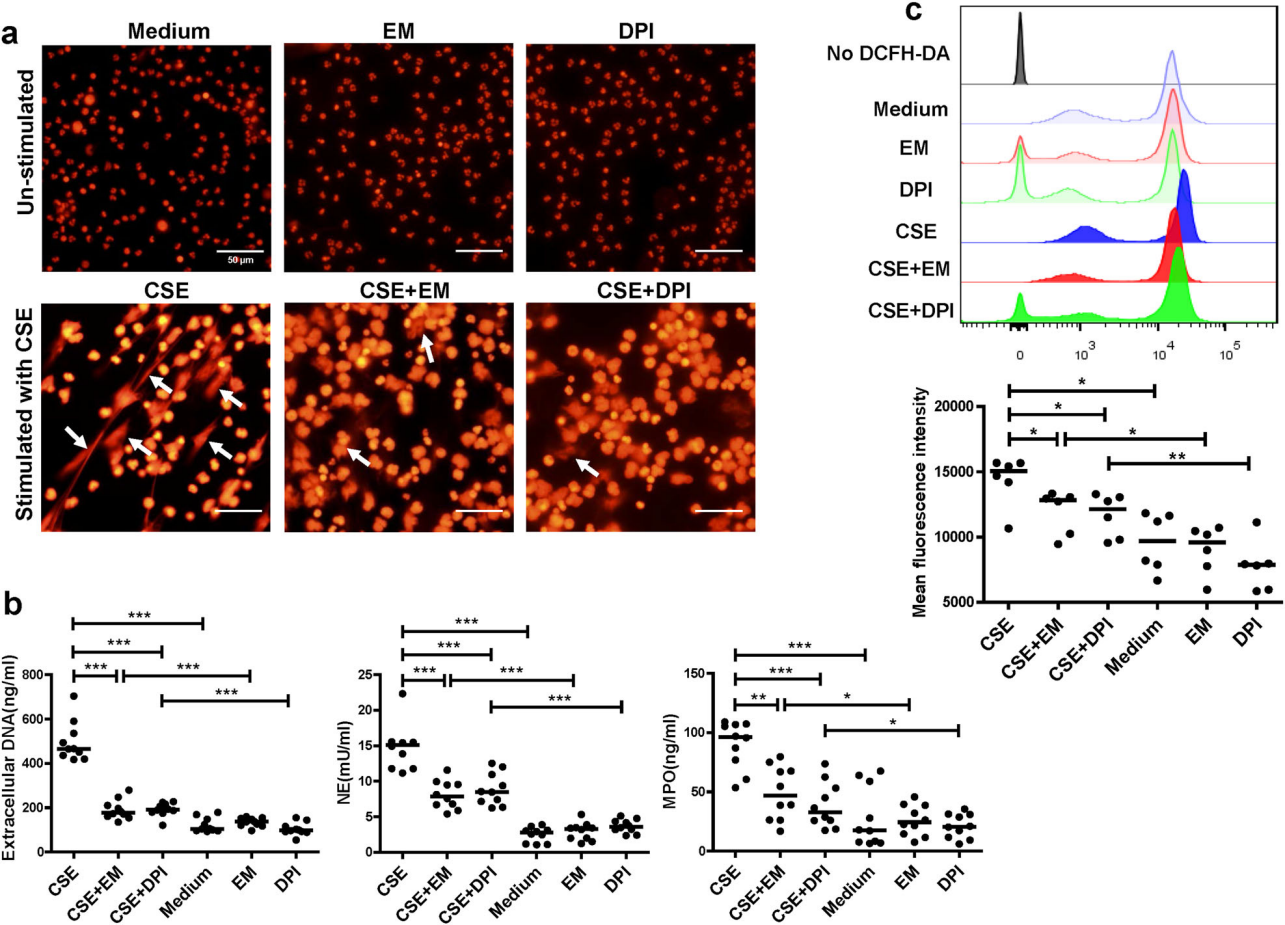
Erythromycin inhibits CSE-induced NETs drived from human. **a** Neutrophils isolated from blood of patients with COPD were stimulated with 0.3% CSE for 4 h at 37 °C ( ± 10 μg/mL erythromycin or ± 5 μg/mL DPI for 30 min). Neutrophils were then fixed with 4% paraformaldehyde for 20 min at room temperature and stained with PI (NETs: white arrows). Original magnification, ×400. Scale bars = 50 µm. **b** PMNs isolated from patients with COPD were stimulated with 0.3% CSE for 4 h at 37 °C ( ± 10 μg/mL erythromycin or ± 5 μg/mL DPI for 30 min). Quantification of extracellular DNA in supernatants was performed by PicoGreen fluorescence assay. Quantification of NET-associated NE and MPO were detected using the NETosis Assay kit and MPO ELISA kit. Data are expressed as medians (*n* = 10). The comparisons were determined by Kruskal–Wallis one-way ANOVA on ranks. *P *<* 0.05, **P *<* 0.01, ***P *<* 0.001. **c** Neutrophils from blood of patients with COPD were pre-treated with erythromycin or DPI for 30 min before CSE stimulation for 1 h. Cells were then stained with 1 μmol/L 2′,7′-dichlorofluorescein diacetate (DCFH-DA) at 37 °C for 30 min and detected by flow cytometry immediately. Intracellular reactive oxygen species (ROS) levels were expressed as mean fluorescence intensity (MFI) of DCFH-DA. Representative histograms of DCFH-DA-dyed PMNs were shown. Quantification of MFI of DCFH-DA were performed by FlowJo v10. The Data are expressed as medians (*n* = 6). The comparisons were determined by Kruskal–Wallis one-way ANOVA on ranks. *P *<* 0.05, **P *<* 0.01

The immunofluorescence results showed that erythromycin- or DPI-pretreated PMNs from COPD patients had fewer filaments (Fig. 6a). Using PicoGreen, we found a lower extracellular DNA concentration in the supernatants of PMNs pre-treated with erythromycin before stimulation, than in PMNs stimulated with CSE alone (*P* *<* 0.001; Fig. 6b). In addition, NET-associated NE and MPO were also decreased in PMNs pre-treated with erythromycin when compared to PMNs stimulated by CSE alone (*P* *<* 0.001 and *P* *<* 0.01; Fig. 6b). Moreover, we observed an increased level of ROS production in PMNs stimulated with CSE (*P* *<* 0.05; Fig. 6c), which could be suppressed by DPI, the inhibitor of phagocyte NADPH oxidase (*P* *<* 0.05; Fig. 6c). Intracellular ROS production was also decreased in PMNs pre-treated with erythromycin when compared to PMNs stimulated by CSE alone (*P* *<* 0.05; Fig. 6c).

**Fig. 7:**
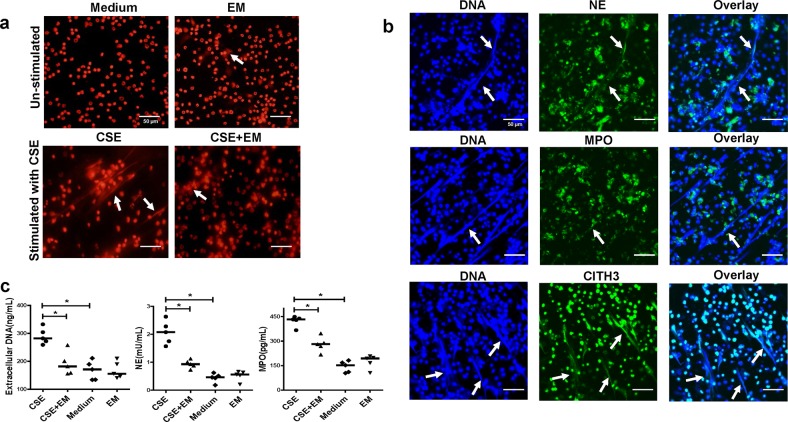
Erythromycin down regulates NET formation of PMNs of cigarette smoke exposed mice in vitro. **a** PMNs isolated from cigarette exposure mice were stimulated with 0.3% CSE for 4 h at 37 °C (±2 μg/mL erythromycin for 30 min). PMNs were then fixed with 4% paraformaldehyde for 20 min at room temperature and stained with PI (NETs: white arrows). Original magnification, ×400. Scale bars = 50 µm. **b** Neutrophils isolated from peripheral blood of cigarette smoke exposed mice were stimulated for NET formation with 0.3% CSE at 37 °C for 4 h. Neutrophils were then fixed with 4% paraformaldehyde. PMNs were stained with NE, MPO or CITH3 (green) and DNA was stained with 4′,6-Diamidino-2-phenylindole dihydrochloride (DAPI, blue), and examined by confocal laser scanning microscope (CLSM) (NETs: white arrows). Original magnification, ×400. Scale bars = 50 µm. **c** The concentrations of extracellular DNA, NET-associated NE and MPO between groups of CSE-stimulated PMNs treated with or without erythromycin. Data are expressed as medians (*n* = 5). The comparisons were determined by Kruskal–Wallis one-way ANOVA on ranks.*P *<* 0.05. NS not significant

For PMNs of mice with emphysema, erythromycin-pretreated PMNs had fewer NETs than those without erythromycin pre-treatment (Fig. 7a). NE, MPO, and CITH3 were observed on CSE-induced NETs by immunofluorescence (Fig. 7b). We found that extracellular DNA as well as NET-associated NE and MPO were lower in the erythromycin-pretreated PMNs than in PMNs stimulated by CSE alone (All *P* *<* 0.05; Fig. 7c).

### Erythromycin inhibits NETs, with amelioration of emphysema, the down-regulation of Th1/Th17 responses and mDCs activation in mice exposed to cigarette smoke

**Fig. 8:**
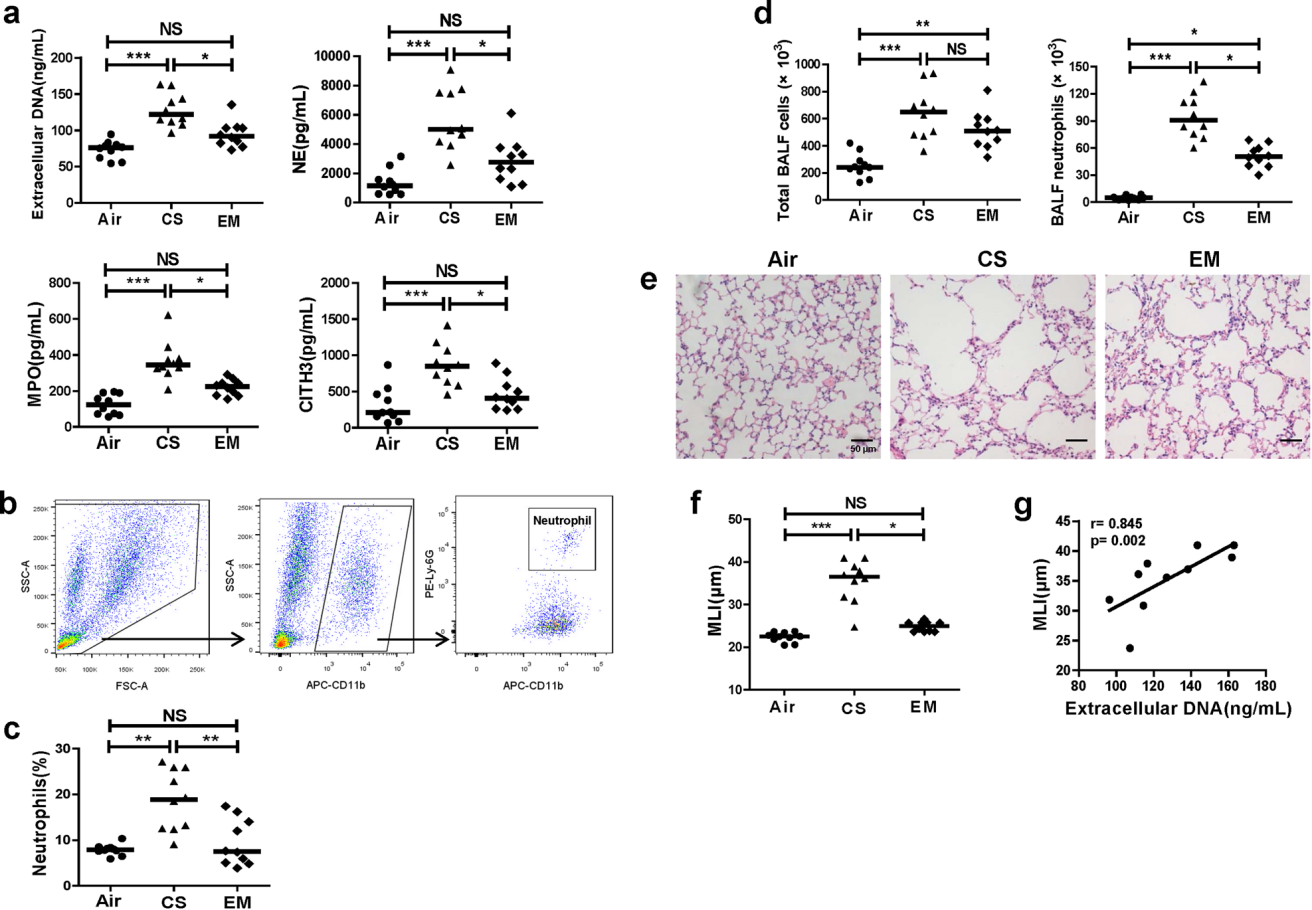
Erythromycin reduces airway NETs and ameliorates emphysema in mice. **a** The extracellular DNA and concentrations of NE, MPO, and CITH3 in BALF supernatants of chronic cigarette smoke exposed mice (CS), cigarette smoke exposed mice treated with erythromycin (EM), and air controls (Air). Data are expressed as medians (*n* = 10). The comparisons were determined by Kruskal–Wallis one-way ANOVA on ranks. *P *<* 0.05, ***P *<* 0.001. NS not significant. **b** Fresh BALF of mice was centrifuged, and the cell pellets were washed twice and finally resuspended in PBS containing 10% fetal bovine serum. The neutrophils in BALF were identified to CD11b^+^Ly-6G^+^ cells by flow cytomertry.The gating strategy was shown. **c** The proportions of neutrophils in CD11b^+^ cells in BALF were detected by flow cytometry. Data are expressed as medians (*n* = 10). The comparisons were determined by Kruskal–Wallis one-way ANOVA on ranks. **P *<* 0.01. NS not significant. **d** The total cell count and neutrophils count in BALF. Data are expressed as medians (*n* = 10). The comparisons were determined by Kruskal–Wallis one-way ANOVA on ranks. *P *<* 0.05,**P *<* 0.01, ***P *<* 0.001. NS not significant. **e** Representative haematoxylin and eosin (H&E) sections of lung tissues from air exposed mice (Air), chronic cigarette smoke exposed mice (CS), and chronic cigarette smoke exposed mice treated with erythromycin (EM). Original magnification ×200. Scale bars = 50 µm. **f** The mean linear intercept (MLI) values in Air group, CS group and EM group was analysed by two independent investigators. Data are expressed as medians (*n* = 10). The comparisons were determined by Kruskal–Wallis one-way ANOVA on ranks. *P *<* 0.05, ***P *<* 0.001. NS not significant. **g** The correlations between extracellular DNA in BALF of cigarette smoke exposed mice and their MLI were determined by Spearman’s rank correlation coefficient

The flow diagram of the experimental design of the mice experiments is shown in Fig. 1b. The mice in the EM group had significantly lower levels of extracellular DNA and soluble NE, MPO, and CITH3 in their BALF than those in the CS group (All *P* *<* 0.05; Fig. 8a). In addition, neutrophils count and the proportions of CD11b^+^Ly-6G^+^ neutrophils in BALF were also decreased in the erythromycin-treated group compared with CS group (Fig. 8b–d). The MLI was lower in the EM group than in the CS group (*P* *<* 0.05; Fig. 8e, f) suggesting a reduction of emphysema in mice treated with erythromycin. Moreover, the MLI of mice with emphysema positively correlated with the levels of extracellular DNA in the BALF (r = 0.845, *P* *=* 0.002; Fig. 8g).

**Fig. 9:**
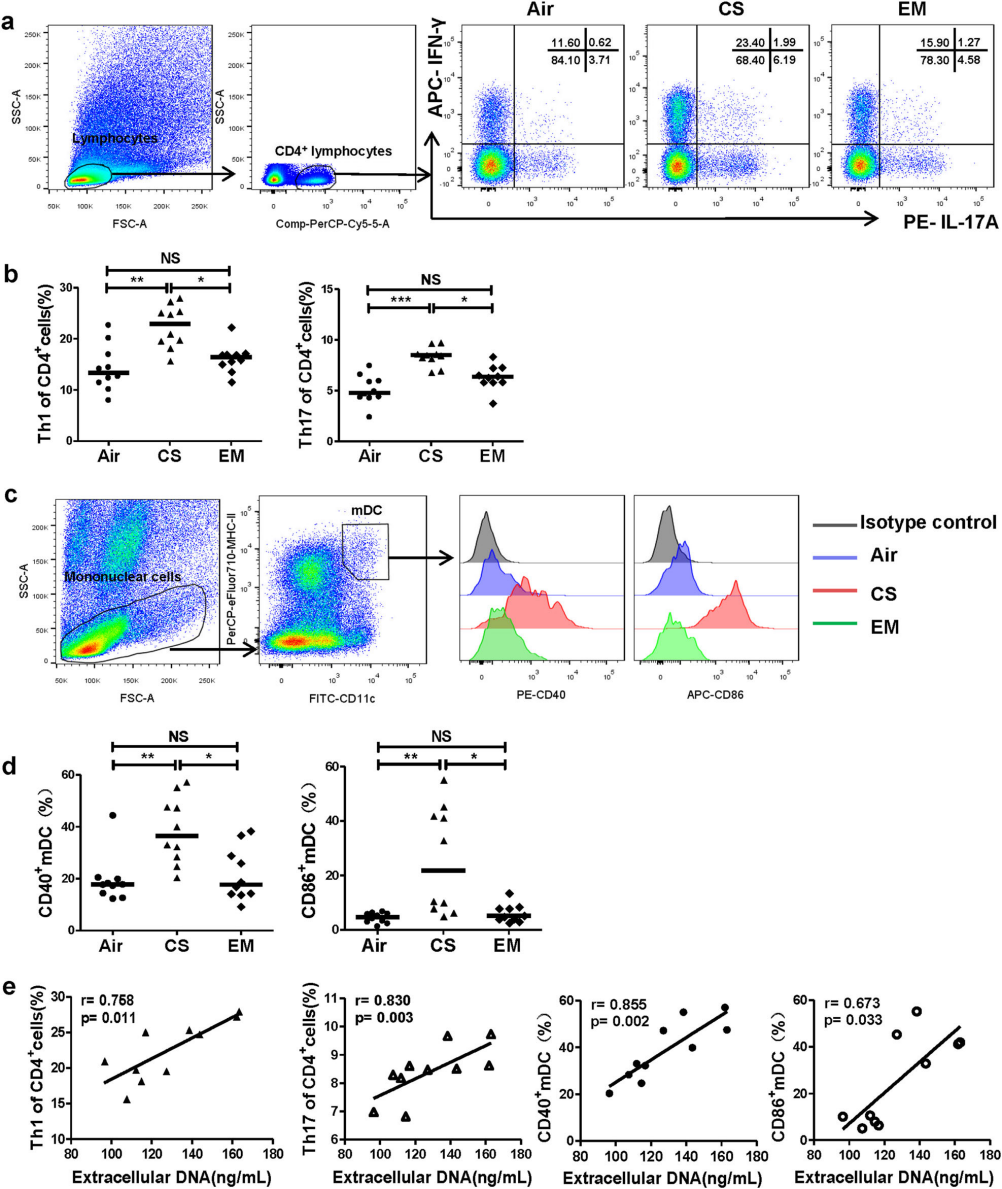
Erythromycin suppresses Th1 and Th17 responses and activation of mDCs along with inhibition of NETs in mice. **a** Lung single-cell suspensions were prepared freshly and detected Th1, Th17 and mDCs by flow cytometry. Lymphocytes of lungs were identified based on FSC and SSC. Th1 cells were identified by CD4^+^IFN-γ^+^ cells, and Th17 cells were identified by CD4^+^IL-17^+^ cells. Representative scatter plots of Th1 and Th17 cells of air controls (Air), cigarette smoke exposed mice (CS) and cigarette smoke exposed mice treated with erythromycin (EM) were shown. **b** The proportions of Th1 and Th17 cells in CD4^+^ cells in lungs of Air group, CS group and EM group. Data are expressed as medians (*n* = 10). The comparisons were determined by Kruskal–Wallis one-way ANOVA on ranks. *P *<* 0.05, **P *<* 0.01, ***P *<* 0.001, NS, not significant. **c** Mononuclear cells of lungs were identified based on FSC and SSC. The mDCs were identified by CD11c^+^MHC-II^high^ cells. Representative histograms of CD40 and CD86 of mDCs in lungs of Air group, CS group and EM group were shown. **d** The proportions of CD40^+^ mDCs and CD86^+^ mDCs in lungs of Air group, CS group and EM group. Data are expressed as medians (*n* = 10). The comparisons were determined by Kruskal–Wallis one-way ANOVA on ranks. *P *<* 0.05, **P *<* 0.01, NS, not significant. **e** The correlations between extracellular DNA in BALF of CS group and their Th1 cells, Th17 cells, CD40^+^ mDCs and CD86^+^ mDCs in lungs were determined by Spearman’s rank correlation coefficient

Furthermore, we observed that mice that treated with erythromycin expressed lower levels of CD40 and CD86 on pulmonary mDCs, along with decreases in Th1 cells and Th17 cells in the lungs than untreated mice (All *P* *<* 0.05; Fig. 9a–d). Moreover, Th1 and Th17 cells, and CD40 and CD86 in the lungs of mice with emphysema positively correlated with the levels of extracellular DNA in BALF (r = 0.758, *P* *=* 0.011; r = 0.830, *P* *=* 0.003; r = 0.855, *P* *=* 0.002; r = 0.673, *P* *=* 0.033; respectively; Fig. 9e).

## Discussion

NET formation can be considered a protective strategy by the host in response to invading pathogens or, in response to tissue damage caused by other harmful environmental irritants. However, an excessive NETs generation may aggravate airway/lung inflammation by eliciting a pathological immune response under conditions of chronic cigarette smoke exposure^16^. Here, our results demonstrated that PMNs from patients with COPD are hypersensitive to cigarette smoke and showed a substantial accumulation of NETs in the airways. NETs induced by CSE promoted mDCs activation, and elicited antigen-specific T-cell responses leading to Th1 and Th17 generation in vitro. We also showed that erythromycin could suppress cigarette smoke-induced NET formation, thus attenuating the pulmonary inflammation in experimental emphysema murine models. Additionally, NETs in mice exposed to cigarette smoke were increased and correlated with severity of emphysema and accompanied with the activation of mDCs and Th1 and Th17 cell responses, which could be suppressed by erythromycin treatment.

Previous studies have reported that NETs in airways were elevated and were positively correlated with airway limitation^26,31^. In agreement with previously published data, neutrophils from smoking COPD patients are hypersensitive to stimulation with CSE with respect to NET formation^32^. However, healthy controls (all non-smokers) did not show increased NETs in the airway. Meanwhile, we found that NETs in sputum did not correlate with smoking pack years. COPD is a highly heterogeneous disorder and there may be differences in the sensitivity in response to cigarette smoke stimulation among individuals. Our finding indicated that NETs in the airway might represent a biomarker of COPD patients.

Chronic neutrophilic inflammation is prominent in the airways of COPD patients^33,34^. However, the development of an effective medication targeting neutrophilic inflammation remains a major challenge. Corticosteroids are commonly used in many chronic airway diseases, but they are not effective for neutrophilic inflammation in COPD. NETs have been proposed as a novel therapeutic target for neutrophilic inflammation in airways including cystic fibrosis, severe asthma and COPD^5,11,35,36^. In this study, erythromycin showed a good inhibitory effect on CSE-induced NETs and NET-associated NE and MPO in vitro, but the mechanism was not so clear. DPI, an inhibitor of the phagocyte NADPH oxidase (NOX2) and NOX2-dependent ROS production, could inhibit CSE-induced NETs with decreased ROS production, which indicated that NOX2-dependent mechanism was probably responsible for NET formation induced by CSE. It has been established that the neutrophil NADPH oxidase activity could be suppressed by erythromycin^37^. Given that erythromycin has a similar effect as DPI, it is very likely that the suppressive capacity of erythromycin on NET formation attributes to suppressing the NOX2. In addition, we found a significant decrease in the neutrophil count in the BALF of erythromycin-treated mice. We also cannot rule out the possibility that erythromycin causes a reduction in extracellular DNA in BALF by decreasing the number of neutrophils. A previous study established that macrolides ameliorate emphysema and reduce airway macrophages, lymphocytes, neutrophils, and pro-inflammatory cytokines in experimental smoking murine models^21^. We extended these previous findings to a mechanistic analysis and found that erythromycin negatively regulated airway neutrophilic inflammation and the release of NETs in the BALF of smoke-exposed mice. Thus, our findings provided direct evidence that erythromycin serves as a potential therapeutic strategy for COPD/emphysema in which neutrophilic inflammation may drive disease progression.

Another perhaps more important consideration is how NETs interact with other components of the immune system and how this affects the processes involved in COPD. This is further emphasised by the observation that inflammation continues in COPD patients after the cessation of smoking. Indeed, high levels of NETs reduce the activation threshold of T cells and DCs.We recently demonstrated that CSE-induced NETs are able to activate plasmacytoid DCs, and then induce CD4^+^T cell polarisation toward Th1 and Th17 phenotypes^5^. In this study, we translated the murine findings to human disease and found that excessive NET formation in sputum is accompanied with over-activated mDCs and aggravated Th1/Th17 cell responses in the blood stream of COPD patients. In vitro, CSE-induced NETs could activate mDCs, and promoted the generation of Th1 and Th17 cells, which showed direct evidence. Further, it was noteworthy that IL-1β, IL-12, and TNF-α released by mDCs were increased with NETs stimulation. TNF-α could promote the maturation of DCs, and IL-12 and IL-1β were involved in CD4^+^ T cell-derived IFN-γ and IL-17 production, respectively^38,39^. CSE-induced NETs is not the only harmful stimulation to the adaptive immune in COPD. For example, CSE could stimulate DCs directly and promote Th17 response^40^. Nonetheless, our findings suggested that NETs may contribute to the activation of the adaptive immune response in COPD, illustrating a unique way in which neutrophils play a synergetic role in this disorder. The down-regulation of NETs under conditions of chronic inflammation induced by cigarette smoke exposure might decrease the activation of the adaptive immune response.

In conclusion, we demonstrated that NETs promote the differentiation of Th1 and Th17 cells and play a role in the adaptive immunity of smoking-related chronic lung inflammation, which indicates that NETs could serve as an important target in COPD. Additionally, cigarette smoke-induced NET formation is inhibited by erythromycin treatment in vitro and in vivo. These findings offer a potential therapeutic strategy for smoking-related COPD/emphysema.

## Supplementary information

Online supplement for full details of methods
