# Supplementary material for: Erythromycin suppresses neutrophil extracellular traps in smoking-related chronic pulmonary inflammation
Source: Cell Death Dis. 2019 Sep 12;10(9):678. doi: 10.1038/s41419-019-1909-2 (PMC6742640; doi:10.1038/s41419-019-1909-2)
Supplement: Supplementary file 1 — Online supplement for full details of methods [file 41419_2019_1909_MOESM1_ESM.docx]

**Erythromycin suppresses neutrophil extracellular traps in smoking-related chronic pulmonary inflammation**

**Authors: Hui Zhang, Shilin Qiu et al.**

**Online supplement for full details of methods**

**Materials & Methods**

**Study Approval**

Thirty-two patients with COPD and sixteen healthy controls were recruited from the First Affiliated Hospital of Guangxi Medical University between May 2017 and May 2018. The diagnosis of COPD was based on forced expiratory volume in 1s (FEV_1_) and forced vital capacity (FVC) detected by post-bronchodilator spirometry (FEV_1_ < 80% predicted and FEV_1_/FVC < 70%) established in the Global Initiative for Chronic Obstructive Lung Disease (GOLD)^1^.The grading of severity of airflow limitation in COPD was defined as: GOLD stage 1: FEV_1_ ≥ 80% predicted；GOLD stage 2: 50% ≤ FEV_1_ < 80% predicted；GOLD stage 3: 30% ≤ FEV_1_< 50% predicted；GOLD stage 4: FEV_1_ < 30% predicted^1^. All patients recruited in this study did not take macrolides within 6 weeks. Human blood and sputum samples were collected after obtaining the approval of the Ethics Committee of the First Affiliated Hospital of Guangxi Medical University (Nanning, China). All patients and control subjects provided written informed consents.

All animal experiments in this study were approved by the Laboratory Animal Ethics Committee of Guangxi Medical University (Nanning, China).

**Human blood and sputum specimen collection and preparation**

Whole blood samples from 32 patients with COPD and 16 healthy control subjects were collected in ethylenediaminetetraacetic acid (EDTA)-treated tubes. Their sputum induction was performed as previously described^2^.

Peripheral blood mononuclear cells (PBMCs) were separated by Lymphoprep (Stemcell Technologies, Canada) centrifugation. Briefly, fresh blood samples were mixed with identical volume of phosphate buffer saline (PBS) and were carefully placed on the surface of Lymphoprep separation medium. After centrifugation at 500×g for 20 min at 28 °C, PBMCs were collected at the interphase and washed with PBS by centrifugation for 10 min at 300×g. Th1 and Th17 cells as well as the costimulatory molecules CD40 and CD86 on myeloid dendritic cells (mDCs) in PBMCs of the 32 patients with COPD and 16 healthy control subjects were analysed by flow cytometry.

PMNs in the blood were isolated by Histopaque 1119 (Sigma-Aldrich, St. Louis, MO, USA) centrifugation to enrich neutrophils and then used Percoll (GE Healthcare, Little Chalfont, UK) density gradient centrifugation, as described previously^3^. The purity of isolated neutrophils was above 95%.

Selected sputum plugs were weighed and divided to two parts. Part of selected sputum plugs was treated with 4 volumes of 0.1% dithiothreitol (DTT; Solarbio Life Sciences, Beijing, China) within 2 h of expectoration. After 20 min of incubation at 37 °C, an identical volume of PBS was added. Subsequently, the cell suspension was filtered through a 53-mm nylon mesh and centrifuged at 4 °C and 1200×g for 10 min. Supernatants were collected and stored at -80 °C. Cell-free extracellular DNA in sputum was detected by using the Quant-iT PicoGreen dsDNA Assay Kit (Invitrogen, Carlsbad, CA, USA). Relative fluorescence was determined using a fluorometer with a filter setting of 480 nm (excitation)/520 nm (emission). The final extracellular DNA concentration was obtained using the standard curve following the manufacturer’s instructions. For immunofluorescence, the other part of selected sputum plugs was diluted with 4 volumes of PBS and incubated at 37 °C for 20 min without adding DTT, and the mixture were used for further processing.

**Animals**

Male C57BL/6J mice (8 weeks old) were purchased from the Guangxi Medical University Laboratory Animal Centre. All mice were housed in sterilized cages in a room maintaining a constant temperature and on a 12-hour light/12-hour dark cycle at Guangxi Medical University Laboratory Animal Center, and all mice received sterilized diet and water. Thirty mice were randomly divided into three groups: Air group, CS group, and EM group (n = 10 per group). The CS group (mice with emphysema induced by cigarette smoke) and EM group (mice with emphysema administered erythromycin) were exposed to cigarette smoke for 24 weeks as previously described^4^. Briefly, mice of CS group and EM group were exposed to five cigarettes (Nanning Zhen long unfiltered cigarettes: 12 mg of tar and 0.9 mg of nicotine) 4 times a day with 30-min smoke-free intervals in a closed 0.75-m^3^ room, 5 days a week, for 24 weeks in total. In the EM group, erythromycin (Sigma-Aldrich, St. Louis, MO, USA) was orally administered at 100 mg/kg/d from the 12th week of cigarette exposure. The vehicle of erythromycin was pure water containing 0.01% dimethylsulfoxide (DMSO; Solarbio Life Sciences) of final concentration. In the CS group and Air group, an identical volume of vehicle was administered orally. Meanwhile, the Air group (mice exposed to air) used as control was exposed to room air for 24 weeks. At the end of the 24th week, 18 h after the last cigarette smoke exposure, mice were anaesthetised with 10% chloralhydrate solution and sacrificed.

Blood samples were collected in EDTA-treated tubes via the posterior ball arteries. PMNs were isolated using peripheral blood neutrophil separation medium for mouse (TBD, Tianjin, China). Briefly, fresh mouse blood samples were carefully placed on the surface of peripheral blood neutrophil separation medium and were centrifuged at 500×g for 20 min at 28 °C. PBMCs were discarded from the upper layer of separation medium. Then the remaining interphases were collected and washed with PBS by centrifugation for 10 min at 300×g. Contaminating erythrocytes were lysed with lysis solution (Solarbio Life Sciences), and PMNs were harvested and resuspended in serum-free RPMI 1640. The purity of the isolated PMNs was above 85%.

Bronchoalveolar lavage fluid (BALF) was collected immediately after mice were anaesthetised. The airways of right lung were lavaged for 4 times through a tracheal cannula with a total of 1 mL of sterilized PBS. Collected BALF was centrifuged at 300×g for 5 min at room temperature. Supernatants were collected and stored at -80 °C. The cell pellet was washed twice and finally resuspended in PBS containing 10% fetal bovine serum (FBS). The total cell count of BALF was performed with a Bürcker chamber and the neutrophil cell count was performed after Wright's Giemsa staining. At least 200 cells were analysed in each sample by two investigators. Flow cytometry analysis was also performed to enumerate CD11b^+^Ly-6G^+^ neutrophils in BALF cells.

The left upper lobe of lung of each mouse was fixed with 10% formalin, paraffin-embedded, sectioned, and stained with haematoxylin and eosin as described previously^5^. The severity of emphysema was assessed by the mean linear intercept (MLI) to estimate the enlargement of alveolar spaces^4^. The right lung and the rest of the left lung were used for the preparation single-cell suspensions by modifying established protocols which contain a combination of mechanical fragmentation, enzyme digestion, and centrifugation procedures as described previously^6^. Firstly, pulmonary circulation was flushed via the right ventricle with PBS to remove the intravascular pool of cells. Then the lungs were minced into 0.1 cm pieces and followed by digestion in 2.5 mg/mL collagenase type IV (Sigma-Aldrich) with RPMI 1640 medium for 45 min at 37 °C. Subsequently, the digested lung tissues were triturated with a plunger from a 5-mL syringe and a 70-μm cell strainer, and then the cell suspension was filtered, washed, followed by red blood cells lysis.

**Cigarette smoke extract (CSE) preparation**

CSE was prepared using a modification of the method as described previously^7^. Briefly, the smoke of 3 cigarettes (Nanning Zhen long unfiltered cigarettes: 12 mg of tar and 0.9 mg of nicotine) was slowly drew into 3 mL of RPMI 1640 medium. The concentration of CSE was determined by using a dual-wave violet spectrophotometer (Lambda Bio 20, Perkin Elmer) at 320 nm absorbance, then the CSE solution was filtered through 0.22-μm filters.

**Immunofluorescence of NETs**

For observation for spontaneous NETs in sputum, part of the selected sputum plugs was diluted with 4 volumes of PBS and incubated at 37 °C for 20 min. Then the diluted sputum was seeded on poly-d-lysine-coated coverslips in 24-well round-bottom culture plates and allowed to settle for 1 h without stimulation. PMNs on coverslips were fixed with 4% paraformaldehyde for 20 min and permeabilized with 0.5% Triton X-100 (Sigma-Aldrich), then blocked (1% bovine serum albumin and 0.1% Tween20; Sigma-Aldrich), and incubated at 4 °C overnight with a Rabbit Anti-Neutrophil elastase (NE) antibody (1:200; Abcam, Cambridge, UK), Rabbit Anti-Myeloeroxidase (MPO) antibody (1:200; Abcam) or Rabbit Anti-Histone H3 (citrulline R2 + R8 + R17) antibody (1:200; Abcam), followed by a Goat polyclonal Secondary Antibody to Rabbit IgG (Alexa Fluor 488) (1:1000; Abcam) for 1 h at 37 °C in the dark. After washing three times with blocking liquid, DNA was stained with 5 μmol/L propidium iodide (PI; Sigma-Aldrich). Slides were washed and mounted with glycerol, then examined by fluorescence microscopy or confocal laser scanning microscope.

For observation for the CSE-induced NETs *in vitro*, PMNs of humans (COPD and healthy controls) or PMNs of mice (CS group and Air group) were seeded on poly-d-lysine-coated coverslips in 24-well round-bottom culture plates (1.0 × 10^6^ cells) with 500 µL of serum-free RPMI 1640 and allowed to settle for 30 min. Then cells were stimulated with 0.3% CSE for 4 h in a CO_2_ incubator at 37 °C with or without pre-treatment with erythromycin (2 μg/mL for mice; 10 μg/mL for humans) for 30 min. In some of the experiments, phorbol-12-myristate-13-acetate (PMA, 100 nmol/L = 61.68 μg /mL; Sigma-Aldrich) and diphenyl iodine (DPI, 5 μg/mL; Sigma-Aldrich) were used as positive controls for stimulating and inhibiting NETs, respectively. PMNs on coverslips were fixed with 4% paraformaldehyde for 20 min and permeabilized with 0.5% Triton X-100 (Sigma-Aldrich). Extracellular DNA was stained with 5 μmol/L PI. In some experiments, for observation for the components of CSE-induced NETs derived from COPD blood or mice with emphysema, after stimulation with 0.3% CSE, fixed with 4% paraformaldehyde and permeabilized with 0.5% Triton X-100, NET components were visualised by staining for NE, MPO, and citrullinated histone H3 (CITH3) with extracellular DNA stained with 5 μmol/L PI or 1 μg/mL 4′,6-diamidino-2-phenylindole dihydrochloride (DAPI; Sigma-Aldrich).

**CSE-induced NET assay**

Freshly isolated PMNs from the blood of COPD or the mice with emphysema were seeded on poly-d-lysine-coated coverslips in 24-well round-bottom culture plates (1.0 × 10^6^ cells) with 500 µL of serum-free RPMI 1640. Subsequently, PMNs were stimulated with 0.3% CSE for 4 h in a CO_2_ incubator at 37 °C, with or without pre-treatment of 30 min with erythromycin (2 μg/mL for mice; 10 μg/mL for humans). Cell-free extracellular DNA in supernatants was detected by using PicoGreen.

For NE and MPO concentration assay, PMNs (2.0 × 10^6^ cells) of patients with COPD or mice with emphysema were seeded in 24-well culture plates and stimulated with 0.3% CSE for 4 h in a CO_2_ incubator at 37 °C with or without pre-treatment of erythromycin for 30 min. After stimulation, supernatant medium was gently aspirated. Wells were washed gently with 1 mL PBS for twice to remove soluble NE and MPO that is not NET–associated. After disruption of NETs with S7 nuclease (15 U/mL; Cayman Chemical, Ann Arbor, MI, USA), EDTA (0.5 mmol/L; Cayman Chemical) was added to stop the reaction. NET-associated NE and were detected by using NETosis Assay kit (Cayman Chemical). NET-associated MPO were detected by using MPO ELISA kit (Cusabio, Wuhan, China) following the manufacturer’s instructions.

For the intracellular reactive oxygen species (ROS) production assay, PMNs (2.0 × 10^6^ cells) of patients with COPD were seeded in 24-well culture plates and stimulated with 0.3% CSE for 1 h in a CO_2_ incubator at 37 °C with or without pre-treatment of erythromycin or DPI for 30min. After stimulation, PMNs were further incubated with 1 μmol/L ROS probe 2′,7′-dichlorofluorescein diacetate (DCFH-DA; Sigma-Aldrich) at 37 °C for 30 min. After the incubation, PBS was added to each tube and then centrifuged at 300×g for 5 min at 4 °C. The supernatants were discarded, and the pellets were resuspended in PBS for intracellular ROS determination by flow cytometry immediately.

**Preparation of monocyte-derived mDCs from PBMCs**

PBMCs were isolated from the blood of healthy donors by Lymphoprep (Stemcell Technologies) centrifugation. Then PBMCs (3.0 × 10^6^ cells/well) were seeded in 6-well culture plates and cultured in RPMI 1640 containing 10% FBS. Four hours later, the supernatants rich of lymphocytes were discarded when all the monocytes settled down and adherent on the bottom of culture plates. Then monocytes were cultured with RPMI 1640 containing 10% FBS, and supplemented 1000 IU/mL granulocyte-macrophage colony stimulating factor (GM-CSF; Peprotech, Rocky Hill, USA) and 500 IU/mL IL-4 (Peprotech) for 6 days as previously described^8^. At day 6, immature mDCs were harvested and isolated by positive selection (CD209 (DC-SIGN) MicroBead Kit, Miltenyi) to obtain mDCs with a purity above 90%.

**Preparation of CSE-induced NETs and stimulation of mDCs**

CSE-induced NETs were prepared as previously described^9^. Briefly, PMNs of patients with COPD were seeded on poly-d-lysine-coated coverslips in 24-well culture plates (1.0 × 10^6^ cells) and stimulated with 0.3% CSE for 4 h in a CO_2_ incubator at 37 °C. All supernatants of stimulation were removed and cells were gently washed 3 times with 1 mL PBS for removing residual CSE. Then the NET-forming cells were treated with restriction enzyme AluI (20 U/mL; TAKALA) in RPMI 1640 for 20 min at 37 °C to obtain soluble NETs. All the supernatants were harvested and centrifuged at 300×g for 5 min at 4 °C to remove contaminating cells and debris. The obtained soluble CSE-induced NETs were quantified by Picogreen.

To determine the effect of CSE-induced NETs on mDCs maturation, the purified immature mDCs of day 6 were cultured in 6-well plates at a density of 1.0 ×10^6^ cells/mL in the presence or absence of CSE-induced NETs (20 ng/mL) for 15 h. Subsequently, cells were harvested and mDCs surface molecules CD11c, CD40, CD86, and HLA-DR were determined by flow cytometry. The supernatants of mDCs were collected and detected the concentrations of IL-1β, IL-12 and TNF-α by ELISA.

**Co-culture of naïve CD4^+^ T cells with mDCs *in vitro***

The purified immature mDCs of day 6 were primed with IFN-γ (10 ng/mL) until day 7. Then the primed mDCs were stimulated with or without CSE-induced NETs (20 ng/mL) for 24 h. CD4^+^ naïve T cells isolated by negative selection (Naive CD4^+^ T Cell Isolation Kit II human, Miltenyi) from PBMCs from healthy donors were cultured with mDCs (pre-treated with or without NETs) at a DC/T cell ratio of 1:5 for 4 days in 24-well plates. In the last 5 h, cells were stimulated with 50 ng/mL PMA (Sigma-Aldrich) and 1 μg/mL ionomycin (Sigma-Aldrich) in the presence of GolgiStop (containing monensin; BD Pharmingen, San Diego, CA, USA). Cells were then harvested and stained for surface marker CD4 followed by intracellular labeling of IFN-γ and IL-17A for flow cytometry.

**Flow cytometry**

For human samples, before evaluating the intracellular IFN-γ and IL-17A, human PBMCs or co-cultured CD4^+^ T cells were stimulated with PMA at 50 ng/mL and 1 μg/mL ionomycin in the presence of GolgiStop at 37 °C in 5% CO_2_ for 5 h. After stimulation, cells were surface-stained with an anti-CD4 monoclonal antibody (Percp-cy5.5; BD Pharmingen) for 30 min at 4 °C and then fixed and permeabilized for 20 min at 4 °C using Fixation/Permeabilization Solution (BD Pharmingen). Subsequently, cells were washed with Perm/Wash buffer (BD Pharmingen), incubated with anti-IFN-γ monoclonal antibody (APC; BD Pharmingen) and anti-IL17A (PE; BD Pharmingen) at 4 °C for 30 min. Th1 cells were identified by CD4^+^IFN-γ^+^ cells and Th17 cells were identified by CD4^+^IL-17A^+^ cells^6^. For detecting mDCs in PBMCs or cultured cells, surface molecules containing CD11c (PE; BD Pharmingen), HLA-DR (PerCP; BD Pharmingen), lineage cocktail I (FITC; BD Pharmingen), CD40 (APC; BD Pharmingen), CD86 (APC; BD Pharmingen) or appropriate isotype controls (BD Pharmingen) were surface-stained for 30 min at 4 °C and fixed with 1% paraformaldehyde. For cultured monocyte-derived cells, CD11c^+^ cells were identified as mDCs, while for PBMCs of human, CD11c^+^HLA-DR^high^Lin^dim/negative^ cells were identified as mDCs^10^.

For mice samples, the Th1 and Th17 cells as well as the costimulatory molecules CD40 and CD86 on mDCs in lungs of Air group, CS group, and EM group were analysed by flow cytometry. To evaluate intracellular cytokines, single-cell suspensions from mouse lungs were stimulated with PMA (25 ng/mL) and ionomycin (1 μg/mL) in the presence of GolgiStop at 37 °C in 5% CO_2_ for 4 h. After stimulation, cells were surface-stained with an anti-CD4 monoclonal antibody (Percp-cy5.5; BD Pharmingen) for 30 min at 4 °C and then fixed and permeabilized for 20 min at 4 °C. Then cells were incubated with anti-IFN-γ monoclonal antibody (APC; BD Pharmingen) and anti-IL17A (PE; BD Pharmingen) at 4 °C for 30 min. Th1 cells were identified by CD4^+^IFN-γ^+^ cells and Th17 cells were identified by CD4^+^IL-17A^+^ cells^6^. For identifying the mDCs of mouse, CD11c (FITC; eBioscence, San Diego, CA, USA), MHC-II (PerCP-eFluor® 710; eBioscence), CD40 (PE; BD Pharmingen), CD86 (APC; BD Pharmingen) or appropriate isotype controls were surface-stained for 30 min at 4 °C and fixed with 1% paraformaldehyde. CD11c^+^MHC-II^high^ cells were identified as mDCs^11^.

Cells of BALF collected from mice were surface-stained by CD11b (APC; eBioscence) and Ly-6G (PE; BD Pharmingen) and fixed with 1% paraformaldehyde. CD11b^+^Ly-6G^+^ cells were identified as neutrophils^12^.

In some experiments, PMNs stained with DCFH-DA were detected by flow cytometry. Intracellular ROS levels were evaluated as mean fluorescence intensity (MFI) of DCFH-DA.

All the prepared samples were analysed by flow cytometry (FAC Canto II; BD Bioscience). FlowJo v10 (Treestar, Ashland, OR, USA) was used to analyse the data.

**ELISA**

The IL-1β, IL-12 and TNF-α concentration in supernatants of mDCs were measured by ELISA with detection limits of 125 pg/mL, 4.7 pg/mL, and 7.8 pg/mL respectively following the manufacturer’s instructions (Cusabio). The soluble NE, MPO and CITH3 levels in BALF of mice were measured by ELISA with detection limits of 0.156 ng/mL, 0.312 ng/mL, and 0.100 ng/mL respectively following the manufacturer’s instructions (NE: USCN Life Sciences, Wuhan, China; MPO: Cusabio; CITH3, Cayman Chemical).

**Statistical analysis**

Results were expressed as medians. Comparisons between two groups were evaluated using Mann-Whitney test. Comparisons between three or more groups were evaluated using Kruskal-Wallis one-way ANOVA on ranks. A correlation analysis was performed using Spearman’s rank correlation coefficient. Analyses were implemented in SPSS version 17.0 and *P* < 0.05 was considered significant.

References

1. Vestbo, J. *et al.* Global strategy for the diagnosis, management, and prevention of chronic obstructive pulmonary disease: GOLD executive summary. *Am J Respir Crit Care Med* **187**, 347-365, doi:10.1164/rccm.201204-0596PP (2013).

2. Grabcanovic-Musija, F. *et al.* Neutrophil extracellular trap (NET) formation characterises stable and exacerbated COPD and correlates with airflow limitation. *Respiratory research* **16**, 59, doi:10.1186/s12931-015-0221-7 (2015).

3. Brinkmann, V., Laube, B., Abu Abed, U., Goosmann, C. & Zychlinsky, A. Neutrophil extracellular traps: how to generate and visualize them. *Journal of visualized experiments : JoVE*, doi:10.3791/1724 (2010).

4. D'Hulst A, I., Vermaelen, K. Y., Brusselle, G. G., Joos, G. F. & Pauwels, R. A. Time course of cigarette smoke-induced pulmonary inflammation in mice. *The European respiratory journal* **26**, 204-213, doi:10.1183/09031936.05.00095204 (2005).

5. Braber, S., Verheijden, K. A., Henricks, P. A., Kraneveld, A. D. & Folkerts, G. A comparison of fixation methods on lung morphology in a murine model of emphysema. *American journal of physiology. Lung cellular and molecular physiology* **299**, L843-851, doi:10.1152/ajplung.00192.2010 (2010).

6. Qiu, S. L. *et al.* Cigarette Smoke Induction of Interleukin-27/WSX-1 Regulates the Differentiation of Th1 and Th17 Cells in a Smoking Mouse Model of Emphysema. *Frontiers in immunology* **7**, 553, doi:10.3389/fimmu.2016.00553 (2016).

7. Moodie, F. M. *et al.* Oxidative stress and cigarette smoke alter chromatin remodeling but differentially regulate NF-kappaB activation and proinflammatory cytokine release in alveolar epithelial cells. *FASEB journal : official publication of the Federation of American Societies for Experimental Biology* **18**, 1897-1899, doi:10.1096/fj.04-1506fje (2004).

8. Hautefort, A. *et al.* T-helper 17 cell polarization in pulmonary arterial hypertension. *Chest* **147**, 1610-1620, doi:10.1378/chest.14-1678 (2015).

9. Qiu, S. L. *et al.* Neutrophil extracellular traps induced by cigarette smoke activate plasmacytoid dendritic cells. *Thorax* **72**, 1084-1093, doi:10.1136/thoraxjnl-2016-209887 (2017).

10. Qiu, S. L. *et al.* Enhanced activation of circulating plasmacytoid dendritic cells in patients with Chronic Obstructive Pulmonary Disease and experimental smoking-induced emphysema. *Clinical immunology (Orlando, Fla.)* **195**, 107-118, doi:10.1016/j.clim.2017.11.003 (2018).

11. Chudnovskiy, A. *et al.* Host-Protozoan Interactions Protect from Mucosal Infections through Activation of the Inflammasome. *Cell* **167**, 444-456.e414, doi:10.1016/j.cell.2016.08.076 (2016).

12. Swamydas, M., Luo, Y., Dorf, M. E. & Lionakis, M. S. Isolation of Mouse Neutrophils. *Current protocols in immunology* **110**, 3.20.21-23.20.15, doi:10.1002/0471142735.im0320s110 (2015).
